# Studies on the Design and Synthesis of Marine Peptide Analogues and Their Ability to Promote Proliferation in HUVECs and Zebrafish

**DOI:** 10.3390/molecules24010066

**Published:** 2018-12-25

**Authors:** Yinglin Zheng, Yichen Tong, Xinfeng Wang, Jiebin Zhou, Jiyan Pang

**Affiliations:** School of Chemistry, Sun Yat-Sen University, Guangzhou 510275, China; zhengylin6@mail2.sysu.edu.cn (Y.Z.); tongych3@mail2.sysu.edu.cn (Y.T.); tomorrow1996@163.com (X.W.); sysuzhoujieb@163.com (J.Z.)

**Keywords:** marine peptides, proliferation, migration, angiogenesis, zebrafish

## Abstract

In our previous studies, tripeptide **1** was found to induce angiogenesis in zebrafish embryos and in HUVECs. Based on the lead compound **1**, seven new marine tripeptide analogues **2**–**8** have been designed and synthesized in this paper to evaluate the effects on promoting cellular proliferation in human endothelial cells (HUVECs) and zebrafish. Among them, compounds **5**–**7** possessed more remarkable increasing proliferation effects than other compounds, and the EC_50_ values of these and the leading compound **1** were 1.0 ± 0.002 μM, 1.0 ± 0.0005 μM, 0.88 ± 0.0972 μM, and 1.31 ± 0.0926 μM, respectively. Furthermore, **5**–**7** could enhance migrations (58.5%, 80.66% and 60.71% increment after culturing 48 h, respectively) and invasions (49.08%, 47.24% and 56.24% increase, respectively) in HUVECs compared with the vehicle control. The results revealed that the tripeptide including l-Tyrosine or d-Proline fragments instead of l-Alanine of leading compound **1** would contribute to HUVECs’ proliferation. Taking the place of the original (l-Lys-l-Ala) segment of leading compound **1**, a new fragment (l-Arg-d-Val) expressed higher performance in bioactivity in HUVECs. In addition, compound **7** could promote angiogenesis in zebrafish assay and it was more interesting that it also could repair damaged blood vessels in PTK787-induced zebrafish at a low concentration. The above data indicate that these peptides have potential implications for further evaluation in cytothesis studies.

## 1. Introduction

Marine peptides are mainly obtained from diverse marine organisms. Marine organisms play an important role as sources of nitrogen and amino acids, which have numerous potential physiological functions [1]. Because of their special marine environment, marine peptides have unique structures, such as rare coded amino acids, special connection bonds and highly modified amino acid residues. The structural diversity of marine peptides results in various bioactivities, such as neurotoxicity [2], anticancer [3], antivirus [4], antimicrobial [5], and antioxidant [6] effects. 

Cellular proliferation is not only one of the most indispensable characteristics of the cell cycle, but the foundation of organism growth, inheritance and evolution. Proliferation plays an important role in physiology and pathology. It is a tightly regulated process and a normal occurrence in numerous biological processes, such as embryogenesis, tissue remodeling, bone development, the ovarian cycle and wound healing [7]. Over the past few years, researchers have started to focus more on cellular proliferation, which has a prominent role to play in the treatment of common diseases. For example, acute dermal wounds heal quickly in healthy individuals but turn into deep sores in diabetics, leading to severe infections in underlying tissues. Therefore, it is vital for promoting faster cellular proliferation and wound healing [8]. In bone repair and regeneration, osteogenic growth peptide (OGP) is a biologically active peptide that affects immune functions, proliferation and differentiation [9]. Furthermore, the significance of promoting proliferation has focused on therapeutic angiogenesis in recent years. Therapeutic angiogenesis, which can re-establish blood perfusion and rescue ischemic tissue, is used to treat ischemic diseases such as peripheral vascular occlusive disease (PVOD), a common manifestation of atherosclerosis with a high rate of morbidity [10]. Zebrafish embryo are recognized as a suitable model to explore the formation of blood vessel because their vascular system can be easily described in the developing embryo. Numerous pathways involved in angiogenesis in mammals are highly conserved in this model.

**Figure 1:**
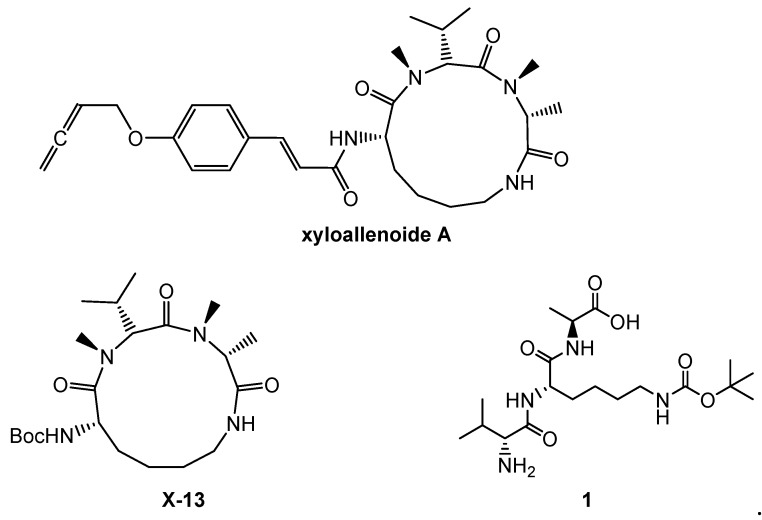
The structures of xyloallenoide A, X-13 and **1**.

In our earlier work, novel marine cyclopeptide analogue xyloallenoide A (Figure 1) was isolated from the mangrove fungus *Xylaria* sp. 2508 in the South China Sea [11]. According to the structure of xyloallenoide A, a *t*-Butyloxy carbonyl (Boc)-protected cyclotripeptide (X-13) was synthesized [12], and it could dose-dependently induce angiogenesis in zebrafish embryos and human umbilical vein endothelial cells (HUVECs), which consisted of Boc-l-Lys, d-N-MeVal and d-N-MeAla. The compound X-13 expressed potent angiogenic properties and is very promising for development as a novel class of pro-angiogenic agents for angiotherapy [13]. Considering the complex structure and hard synthesis of cyclopeptides, a series of linepeptides were designed and synthesized [13]. Among them, tripeptide **1** with the group of d-Val, Boc-l-Lys and l-Ala had the strongest induced angiogenesis effect, both in vivo and in vitro. The effect of tripeptide **1** on angiogenesis wasmore significant than that of the compound X-13 [14]. Previous structure–activity relationship (SAR) analysis revealed that linear tripeptides and tetrapeptides, including Val, Lys and Ala amino acid segments, displayed favorable activities.

**Figure 2:**
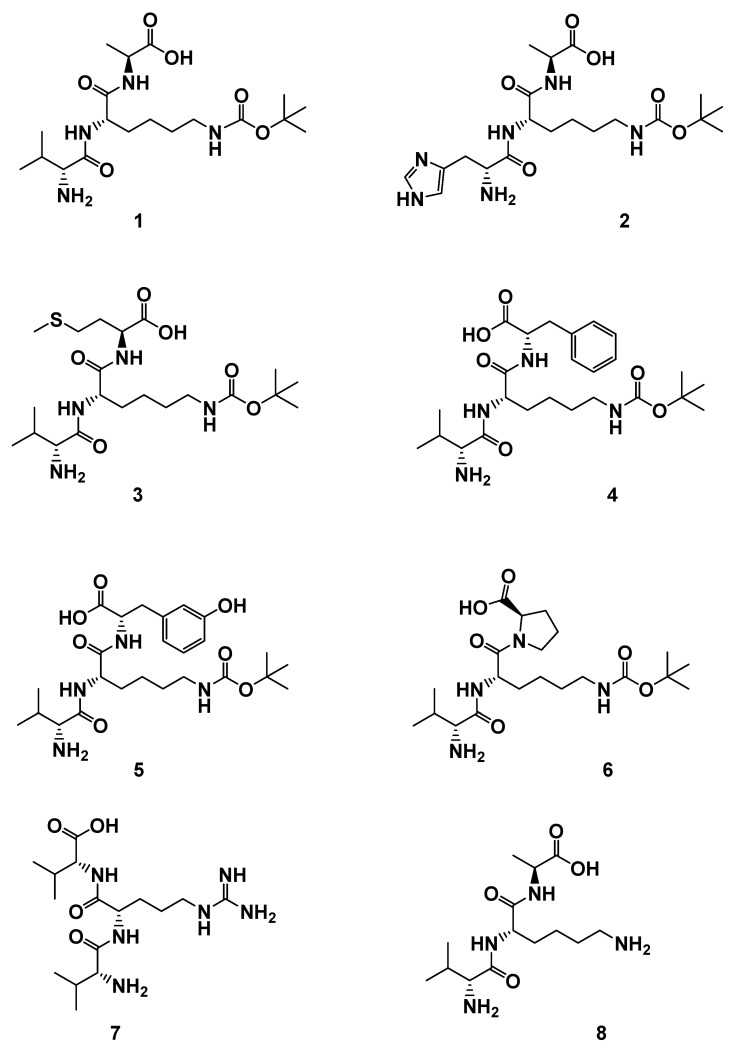
The structures of derivatives **1**–**8**. H-d-Val-l-Lys(Boc)-l-Ala-OH (**1**); H-d-His-l-Lys(Boc)-l-Ala-OH (**2**); H-d-Val-l-Lys(Boc)-l-Met-OH (**3**); H-d-Val-l-Lys(Boc)-l-Phe-OH (**4**); H-d-Val-l-Lys(Boc)-l-Tyr-OH (**5**); H-d-Val-l-Lys(Boc)-d-Pro-OH (**6**); H-d-Val-l-Arg-d-Val-OH (**7**); H-d-Val-l-Lys-l-Ala-OH (**8**).

In this paper, to explore more leading bioactive compounds resembling compound **1**, we designed a series of new tripeptides. Based on lead compound **1**, more tripeptides **2**–**8** (Figure 2), including a variety of different amino acids and substituents, were synthesized. Further promoting cellular proliferations were performed on HUVECs to identify more new candidate drugs and discuss the SAR. Moreover, the further proliferative and angiogenesis effects of selected compound were evaluated on normal and damaged zebrafish models.

## 2. Results and Discussion

### 2.1. Chemistry

To assess the cellular bioactivities of different modifications of compound **1**, a series of analogues **2**–**8** were designed, with modification focused on the different amino acids and the lipophilic/hydrophilic and acidity/alkaline properties of compounds. With the aim of studying the steric effect, compounds **4** and **8** were designed. Compound **2** was synthesized to explore the bioactivity of unsaturated alkaline amino acid, with d-Histidine instead of d-Valine. Compounds **3** and 6 were focused on the effect of the structure of the methylthio group and tetrahydropyrrole on cellular bioactivities. Furthermore, acidity and hydrophily are probably related to activity, so compound **5** was designed. Due to the Arg-Gly-Asp (RGD) sequence relating to angiogenesis [15], compound **7** containing an RGD moiety was synthesized.

**Scheme 1:**
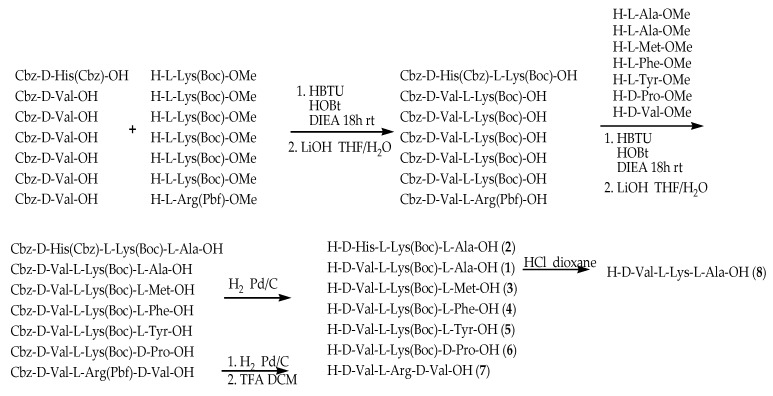
Synthetic route of compounds **1**–**8**. All peptides were synthesis by HBTU, HOBt and DIEA as coupling reagents at room temperature 18 h. The dipeptide and tripeptide were demethylated using LiOH in THF/H_2_O. All Cbz-groups were removed by H_2_ with Pd/C-catalyzed.

The line peptide compounds **1**–**8** were prepared by our previous method [12] (Scheme 1). Generally, Cbz-d-Val-OH, Cbz-d-His-OH, H-l-Lys(Boc)-OMe and H-l-Arg(Pbf)-OMe were used as starting materials and coupled with another amino acid by coupling reagents (HOBt, HBTU and DIEA) to obtain the corresponding dipeptide and tripeptide. The dipeptide and tripeptide were demethylated using LiOH in THF/H_2_O. All Cbz-groups were removed by H_2_ with Pd/C-catalyzed. All the target molecules were purified through flash column chromatography, and the structures were fully characterized by ^1^H NMR, ^13^C NMR and HR-EI-MS. The purity of all target compounds was ≥95% as determined by HPLC analysis.

### 2.2. Effects of Compounds ***1***–***8*** on HUVEC Proliferation

**Figure 3:**
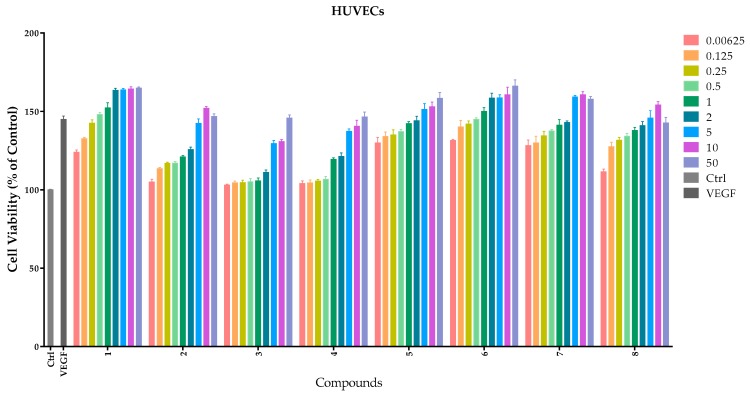
Effects of compounds **1**–**8** on proliferation of HUVECs. HUVECs were cultured with different concentrations (0–50 μM) of compounds. Cellular proliferation was assessed using the thiazolyl blue tetrazolium bromide (MTT) assay after 48 h. Data are expressed as the mean ± SEM (*n* = 4) of three individual experiments. The x-axis represents different compounds and the y-axis represents the cell viability (the control as 100%); different column colors represent different concentrations from 0.0625 μM to 50 μM.

The endothelial cell’s proliferation is an important phase in the process of normal life. Human umbilical vein endothelial cells (HUVECs) are frequently used to measure the angiogenic property in vitro. HUVECs are usually used as a laboratory model system for the study of the function and pathology of endothelial cells such as angiogenesis [16] and hypertension [17]. Like human umbilical artery endothelial cells, they exhibit a cobblestone phenotype when lining vessel walls. To evaluate the cellular bioactivity in vitro, compounds **1**–**8** were studied on the HUVECs with different concentrations: 0.0625 μM, 0.125 μM, 0.25 μM, 0.5 μM, 1 μM, 2 μM, 5 μM, 10 μM, and 50 μM. A quantity of 20 ng/mL VEGF was used as a positive control. The results are shown in Figure 3 and Table 1.

Lead compound **1** clearly showed a notable proliferative effect on HUVECs with EC_50_ value of 1.3 ± 0.0926 μM in a concentration-dependent manner. Compounds **5**–**7** possessed better proliferation effects with respect to HUVECs than other compounds, and the EC_50_ values were 1.0 ± 0.002 μM, 1.0 ± 0.0005 μM and 0.88 ± 0.0972 μM, respectively.

In view of the assays of promoting proliferation, the SAR analysis revealed that:

(a) the D-histidine fragment of compound **2** replaced the d-valine of compound 1, or L-methionine fragment of compound **3** replaced the L-alanine fragment of compound 1; both reduced cellular bioactivity, which might indicate that cellular proliferation was depressed due to steric hindrance of the substrates;

(b) secondly, the l-Tyrosine with phenol group (compound **5**) or d-proline with tetrahydropyrrole fragment (compound **6**) substituting for L-alanine also increased proliferation in HUVECs probably through hydrogen-bonding interaction; 

(c) to our surprise, compound **7** exerted strong effects on HUVECs, which revealed that the l-Arg-d-Val fragment resembles the Lys(Boc)-l-Ala-OH in cytoactive terms; 

(d) compared with compound **1** and **8**, we found that the *t*-Butyloxy carbonyl group was not a determinant factor in increasing cellular proliferation of HUVECs.

### 2.3. Migration Assays–Wound Healing of Compounds ***5***–***7***

**Figure 4:**
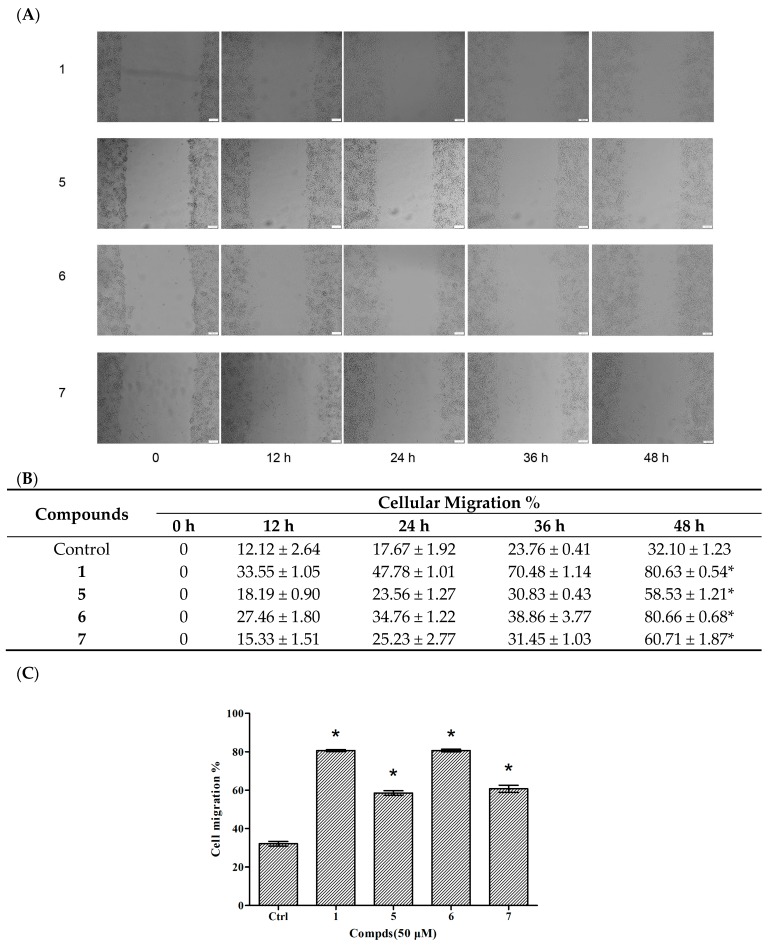
Effects of compounds **1** and **5**–**7** on HUVEC migration. (**A**) Observation of the effect of compounds on HUVEC migration; (**B**) The values of the compound-induced HUVEC migration at 0–48 h post-wounding; (**C**) Quantitative evaluation of the migration of HUVECs. Cellular migration was assessed at 48 h post-wounding. Data are expressed as the mean ± SEM (*n* = 4) of three individual experiments. Values vs control group: * *p* < 0.01 versus control.

Cellular migration is a central process in the development of multicellular organisms. The wound healing method was used to evaluate the effects of compounds on HUVEC migration. Based on the results of proliferative assay on HUVECs, compounds **1** and **5**–**7** were chosen to evaluate the effects of reconstruction and migration at 50 μM. DMSO served as a control. The states of cellular growth at 0 h, 12 h, 24 h, 36 h and 48 h are presented in Figure 4. No significant endothelial cellular migrations were found in compound-treated groups and the vehicle control HUVEC before 12 h. However, compounds **1**, **5**, **6** and **7** treated groups all showed an increase in migration (58.5%, 80.66%, 60.71%, and 80.63% increment, respectively) after 48 h when compared with the vehicle control.

### 2.4. Invasion Assays of Compounds ***5***–***7*** in HUVECs

**Figure 5:**
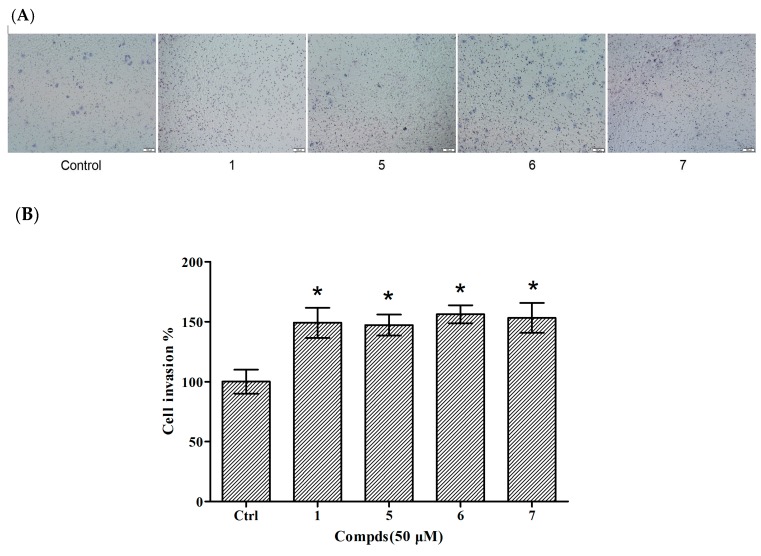
Effects of compounds **1** and **5**–**7** on HUVEC invasion. (**A**) Observation of the effect of compounds on HUVEC invasion; (**B**) Quantitative evaluation of the compound-induced HUVEC invasion. Cellular invasion was assessed at 24 h. Data are expressed as the mean ± SEM (*n* = 3) of three individual experiments. Values vs control group: * *p* < 0.01 versus control.

Cellular proliferation, migration and invasion are clear characteristics of cytothesis in organisms. Therefore, transwell assays were utilized to determine the invasion of compounds **1** and **5**–**7** in HUVECs. There were 49.08%, 47.24%, 56.24%, and 53.17% increases in the invasion of HUVECs treated with compound **1** and **5**–**7** at 50 μM, respectively (Figure 5). The results indicated that compounds **5**–**7** were capable of inducing HUVEC migration similar to compound **1**. The above results suggest that these three tripeptides possess potential in the application of cytothesis studies.

### 2.5. The Angiogenic Activity of Compound ***6*** in Zebrafish

**Figure 6:**
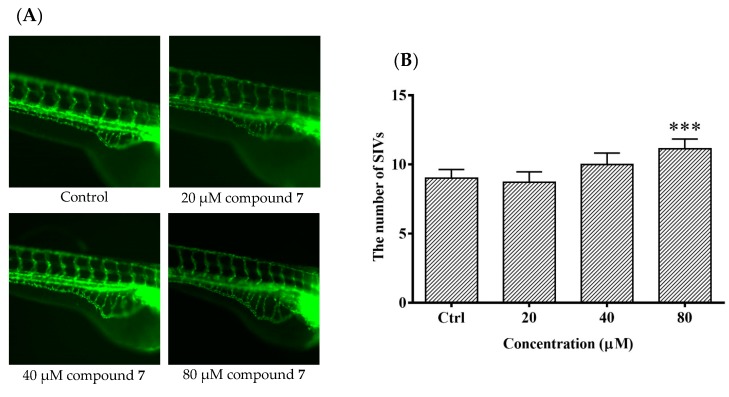
The effects of compound **7** on the angiogenesis formation in transgenic Tg (fli1: EGFP) zebrafish. Zebrafish embryos (24 hpf) were treated with test solution for 48 h and were evaluated using a microscope. (**A**) Representative images of blood vessel formation of zebrafish larvae at 72 hpf; (**B**) Quantitative analysis of the number of subintestinal vessel plexus (SIVs). Data are expressed as the means ± SEM (*n* = 10), and statistical significance was assessed by one-way ANOVA. Values vs control group: *** *p* < 0.001.

**Figure 7:**
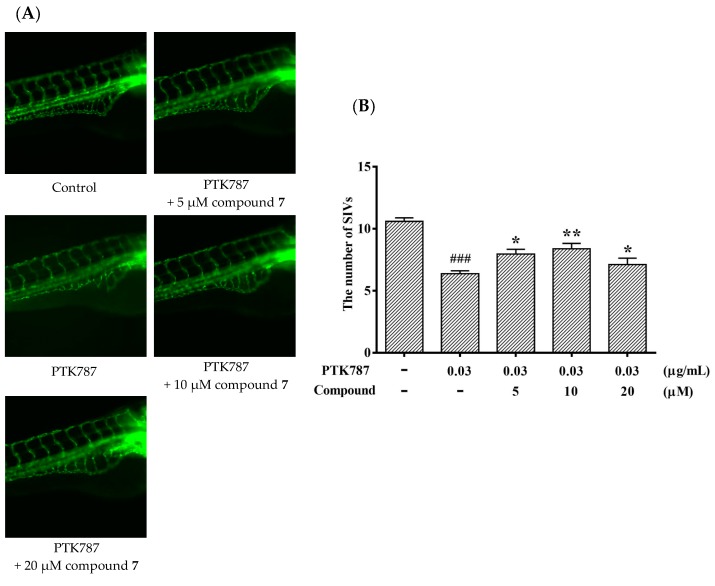
Compound **7** relieved the injuries of damaged SIVs in zebrafish. Zebrafish embryos (24 hpf) were treated with PTK787 for 24 h, then continually incubated with mixed solution of PTK787 and compound until 72 hpf. Zebrafish embryos were evaluated using a microscope. (**A**) Representative images of blood vessel formation of zebrafish larvae at 72 hpf; (**B**) Quantitative analysis of the regenerating caudal fin. Data are plotted as the mean ± SEM (*n* = 20), and statistical significance was assessed by one-way ANOVA. Values vs control group: ^###^ *p* < 0.001; values vs model group: * *p* < 0.05 and ** *p* < 0.01.

It is meaningful to explore new candidate drugs for angiogenic therapy. An increasing number of studies are now available on the zebrafish model due to its short life cycle, availability and low cost. Based on the above assays, the proliferative effect of compound **7** was the most of significant on HUVEC proliferation. To further explore the effect of angiogenesis and restoration of blood vessel injury of compound **7**, the zebrafish assay was performed. The angiogenesis effects of compound **7** on normal zebrafish and PTK787-induced zebrafish blood vessel injury are presented in Figure 6 and Figure 7, respectively. The results indicated that compound **7** could promote angiogenesis in zebrafish. It was more interesting that compound **7** could relieve the injuries of damaged sub-intestinal vein (SIV) on PTK787-induced zebrafish at a low concentration at 5 μM (*p* < 0.05), indicating that it can repair damaged blood vessels.

## 3. Experimental Section

### 3.1. Chemistry

All reagents and solvents were of commercial quality. NMR data were recorded in methanol or DMSO, using TMS as an internal reference on a Varian Inova 500 MB NMR spectrometer (^1^H, 500 MHz; ^13^C, 125 MHz, Varian Medical Systems, Inc., Palo Alto, CA, USA), Bruker Avance 400 MB NMR spectrometer (^1^H, 400 MHz; ^13^C, 101 MHz, Bruker Corporation, Billerica, MA, USA). HREIMS were measured using Thermo MAT95XP High Resolution mass spectrometry (Thermo Fisher Scientific Inc. Waltham, MA, USA). EI were recorded on a Thermo DSQ EI-mass spectrometer. Column chromatography was carried out on silica gel (200–300 mesh, Qingdao Haiyang Chemical Co. Ltd., Qingdao, China). High-performance liquid chromatography (HPLC) was performed on a, Shimadzu LC-2010c (Shimadzu Corporation, Kyoto, Japan) equipped with UV detector. The purity of all compounds synthesized in this study was ≥95% as determined by HPLC analysis. Compounds **2**–**8** were first reported. The HPLC of compounds were shown in the Supplementary Flies (Tables S1–S9).

### 3.2. General Procedure for the Synthesis of Compounds ***1***–***8***

All the tested compounds were synthesized according to the literature [12]. Generally, amino acids Cbz-d-Val-OH and H-l-Lys(Boc)-OMe were used to starting materials and HBTU, HOBt and DIEA as coupling reagents. The mixtures were dissolved in DCM and stirred for 18 h at room temperature, followed by demethylation to form Cbz-d-Val-l-Lys(Boc)-OH. Coupling Cbz-d-Val-l-Lys(Boc)-OH and the O-methylation of L-alanie, L-methionine, L-phenylanaline, L-typrosine, and D-proline, respectively, in the same way and then demethylating with LiOH in THF/H_2_O to form compounds **1**, **3**, **4**, **5** and **6**. Tripeptide **8** was obtained by removal of the Boc group of compound **1** in HCl/dioxane. Similarly, tripeptide **2** was obtained by coupling Cbz-d-His(Cbz)-OH, H-l-Lys(Boc)-OMe and H-l-Ala-OMe. Compound **7** was prepared by coupling Cbz-d-Val-OH, H-l-Arg(Pbf)-OMe and H-d-Val-OMe. The intermediate was then stirred in TFA/DCM to remove the Pbf-group to yield tripeptide 7. The NMR spectrum of compounds were shown in the Supplementary Flies (Figures S1–S15).

*H-d-Val-l-Lys(Boc)-l-Ala-OH* (**1**). White solid. ^1^H NMR (500 MHz, MeOD) δ 8.54 (d, *J* = 8.2 Hz, 1H), 8.37 (d, *J* = 7.3 Hz, 1H), 6.68 (s, *J* = 8.4, 5.5 Hz, 1H), 4.21 (p, *J* = 7.4 Hz, 1H), 3.68 (d, *J* = 5.2 Hz, 1H), 2.88 (dd, *J* = 13.1, 6.7 Hz, 2H), 2.51 (dt, *J* = 3.6, 1.8 Hz, 7H), 2.18–1.98 (m, 1H), 1.73–1.45 (m, 2H), 1.30 (s, 11H), 1.29 (d, *J* = 7.3 Hz, 4H), 0.94 (dd, *J* = 11.8,6.9 Hz, 6H). ^13^C NMR (125 MHz, MeOD) *δ* 174.26, 171.33, 168.10, 156.00, 77.81, 57.78, 52.63, 47.88, 30.35, 29.52, 28.74, 22.94, 18.86, 17.82, 17.52. EI-MS: *m*/*z* 417.5 (M^+^); HR-EI-MS calcd. for C_19_H_36_O_6_N_4_: 417.5257 (M^+^), found: 417.5228.

*H-D-His-l-Lys(Boc)-l-Ala-OH* (**2**). White solid. ^1^H NMR (500 MHz, MeOD) δ 8.72 (s, 1H), 7.38 (s, 1H), 4.42–4.34 (m, 1H), 4.31 (dd, *J* = 8.7,5.3 Hz, 1H), 4.17 (t, *J* = 7.1 Hz, 1H), 3.25 (m, *J* = 15.0, 6.6 Hz, 1H), 3.00 (t, *J* = 7.2 Hz, 2H), 1.77 (dt, *J* = 13.8, 7.4 Hz, 1H), 1.69–1.58 (m, 1H), 1.40 (d, *J* = 7.3 Hz, 4H). ^13^C NMR (125 MHz, MeOD) *δ* 174.25, 172.15, 167.32, 161.62, 157.30, 134.85, 128.41, 117.50, 110.01, 53.35, 52.24, 39.74, 31.43, 29.22, 27.38, 26.83, 22.58, 16.05. EI-MS: *m*/*z* 455.10 (M^+^); HR-EI-MS calcd. for C_20_H_35_O_6_N_6_: 455.2612 (M^+^), found: 455.2611.

*H-d-Val-l-Lys(Boc)-l-Met-OH* (**3**). White solid. ^1^H NMR (500 MHz, MeOD) δ 4.59 (dd, *J* = 9.5, 4.3 Hz, 1H), 4.34 (dd, *J* = 9.0, 5.2 Hz, 1H), 3.82 (d, *J* = 3.6 Hz, 2H), 3.80 (d, *J* = 3.5 Hz, 3H), 3.77 (d, *J* = 7.8 Hz, 5H), 3.73–3.67 (m, 5H), 3.67–3.56 (m, 6H), 3.10–2.98 (m, 2H), 2.63 (ddd, *J* = 13.5, 8.5, 5.0 Hz, 1H), 2.53 (dt, *J* = 13.5, 7.9 Hz, 1H),2.23–2.13 (m, 2H), 2.09 (s, 4H), 1.96 (ddd, *J* = 14.2, 8.8, 4.3 Hz, 1H), 1.87 (dt, *J* = 12.7, 6.1 Hz, 1H), 1.77–1.63 (m, 1H), 1.45 (m, *J* = 55.4, 14.3, 7.2 Hz, 16H), 1.06 (dd, *J* = 6.8, 5.1 Hz, 7H). ^13^C NMR (125 MHz, MeOD) *δ* 174.81, 174.47, 169.67, 158.59, 79.93, 73.03, 71.40, 65.15, 59.87, 54.90, 52.21, 32.76, 32.19, 31.42, 31.18, 30.54, 28.79, 24.25, 19.03, 18.03, 15.11. EI-MS: *m*/*z* 477.05 (M^+^); HR-EI-MS calcd. for C_21_H_39_O_6_N_4_S: 475.2595 (M^−^), found: 475.2597.

*H-d-Val-l-Lys(Boc)-l-Phe-OH* (**4**). White solid. ^1^H NMR (500 MHz, MeOD) *δ* 7.32–7.24 (m, 1H), 7.23–7.14 (m, 1H), 4.63 (dd, *J* = 8.2, 5.2 Hz, 1H), 4.34 (dd, *J* = 9.1, 4.8 Hz, 1H), 3.63 (d, *J* = 6.2 Hz, 1H), 3.19 (dd, *J* = 14.0, 5.3 Hz, 1H), 3.02 (ddd, *J* = 8.4, 7.4, 4.4 Hz, 1H), 2.17 (dq, *J* = 13.6, 6.8 Hz, 1H), 1.8 (ddd, *J* = 14.3, 10.8, 5.9 Hz, 1H), 1.73–1.60 (m, 1H), 1.51–1.32 (m, 3H), 1.05 (t, *J* = 7.4 Hz, 1H ). ^13^C NMR (125 MHz, MeOD) *δ* 173.14, 172.67, 168.12, 157.18, 136.98, 128.98, 128.05, 126.39, 78.51, 88.49, 53.99, 53.38, 39.66, 36.85, 31.57, 29.99,29.12, 27.38, 22.84, 17.65, 16.55. EI-MS: *m*/*z* 493.10 (M^+^); HR-EI-MS calcd. for C_25_H_39_O_6_N_4_: 491.2875 (M^−^), found: 491.2877.

*H-d-Val-l-Lys(Boc)-l-Tyr-OH* (**5**). White solid. ^1^H NMR (500 MHz, MeOD) δ 7.07 (d, *J* = 8.5 Hz, 1H), 4.56 (dd, *J* = 8.0, 5.1 Hz, 1H), 4.34 (dd, *J* = 9.0, 4.5 Hz, 1H), 3.86–3.73 (m, 9H), 3.73–3.67 (m, 4H), 3.66–3.57 (m, 5H), 3.14–2.80 (m, 2H), 2.17 (dd, *J* = 13.4, 6.7 Hz, 1H), 1.80 (dd, *J* = 14.3, 8.2 Hz, 1H), 1.72–1.61 (m, 1H), 1.05 (dd, *J* = 8.3, 7.1 Hz, 3H). ^13^C NMR (125 MHz, MeOD) *δ* 155.92, 150.16, 129.99, 114.80, 71.63, 70.00, 63.75, 58.50, 54.25, 53.39, 36.11, 31.59, 29.99, 29.13, 27.38, 22.84, 17.66, 16.54. EI-MS: *m*/*z* 509.05 (M^+^); HR-EI-MS calcd. for C_25_H_39_O_7_N_4_: 507.2824 (M^−^), found: 507.2825.

*H-d-Val-l-Lys(Boc)-d-Pro-OH* (**6**). White solid. ^1^H NMR (500 MHz, MeOD) δ 5.05 (t, *J* = 5.5 Hz, 1H), 4.71 (dd, *J* = 8.9, 4.9 Hz, 2H), 4.47–4.39 (m, 2H), 4.35 (dd, *J* = 9.7, 4.2 Hz, 1H), 3.91 (dd, *J* = 10.8, 6.0 Hz, 2H), 3.64 (dt, *J* = 9.5, 4.7 Hz, 4H), 3.49 (ddd, *J* = 19.1, 11.3, 6.0 Hz, 1H), 3.10–2.95 (m, 4H), 2.32–2.24 (m, 2H), 2.22–2.14 (m, 2H), 2.11–2.00 (m,5H), 1.94 (dd, *J* = 15.0, 7.6 Hz, 1H), 1.8 (dd, *J* = 16.2, 7.0 Hz, 2H), 1.67 (ddd, *J* = 14.0, 11.6, 6.8 Hz, 2H), 1.13 0 0.97 (m, 14H). ^13^C NMR (125 MHz, MeOD) δ 173.93, 171.00, 167.96, 157.19, 78.48, 59.62, 59.22, 58.49, 58.31, 39.50, 30.57, 29.14, 28.83, 27.38, 24.20, 22.74, 22.03, 17.69, 16.54. EI-MS: *m*/*z* 443.05 (M^+^); HR-EI-MS calcd. for C_21_H_37_O_6_N_4_: 441.2718 (M^−^), found: 441.2720.

*H-d-Val-l-Arg-d-Val-OH* (**7**)**.** White solid. ^1^H NMR (500 MHz, MeOD) δ 4.58 (dd, *J* = 7.9, 6.0 Hz, 1H), 4.32 (d, *J* = 5.6 Hz, 1H), 3.85–3.77 (m, 2H), 3.77 (s, 1H), 3.69 (q, *J* = 5.7 Hz, 2H), 3.63 (dd, *J* = 11.1, 5.9 Hz, 1H), 3.26–3.14 (m, 3H), 2.19 (dqd, *J* = 13.7, 6.9, 4.0 Hz, 2H), 1.94–1.81 (m, 1H), 1.81–1.53 (m, 4H), 1.06 (dd, *J* = 8.6, 7.0 Hz, 7H), 0.98 (dd, *J* = 6.8, 4.2 Hz, 8H). ^13^C NMR (125 MHz, MeOD) *δ* 171.95, 168.26, 157.24, 71.63, 70.01, 63.75, 58.43, 57.90, 40.56, 30.39, 30.03, 29.42, 25.21, 16.29, 17.60, 16.54. EI-MS: *m*/*z* 373.10 (M^+^); HR-EI-MS calcd. for C_16_H_33_O_4_N_6_: 373.2557 (M^+^), found: 373.2557.

*H-d-Val-l-Lys-l-Ala-OH* (**8**)**.** White solid. ^1^H NMR (400 MHz, MeOD) δ 4.41 (dd, *J* = 11.8, 6.0 Hz, 1H), 3.71 (d, *J* = 6.2 Hz, 1H),2.92 (t, *J* = 7.5 Hz, 1H), 2.20 (dq, *J* = 13.4, 6.6 Hz, 1H), 1.90 (tq, *J* = 14.0, 7.4 Hz, 1H), 1.74 (ddd, *J* = 23.2, 15.0, 7.8 Hz, 2H), 1.54 (dd, *J* = 15.0, 7.5 Hz, 1H), 1.39 (dd, *J* = 36.4, 19.5 Hz, 2H), 1.16–0.97 (m, 3H). EI-MS: *m*/*z* 317.20 (M^+^); HR-EI-MS calcd. for C_14_H_29_O_4_N_4_: 317.2183 (M^+^), found: 317.2178.

### 3.3. Cellular Culture and Drug Treatment

Human umbilical vein endothelial cellular (HUVEC) cells were obtained from ScienCell Research Laboratories, Inc. (San Diego, CA, USA). (CAT. 8000). HUVECs were cultured in M199 medium with 100 μg/mL penicillin-streptomycin, 30 μg/mL endothelial cellular growth supplement and 10% FBS in 75 cm^2^ tissue culture flasks at 37 °C in a humidified atmosphere of 5% CO_2_. Compounds were dissolved in DMSO to make a 200 μM stock solution and were then diluted to different concentrations as needed.

### 3.4. Proliferative Assays

HUVECs were seeded onto 96-well gelatin coated plates at a density of 10^4^ cells/well. In order to achieve a quiescent state, complete medium was replaced after 24 h incubation with low serum (0.5% FBS) medium and re-incubated for 24 h. After this, the medium was replaced with various drug treatments diluted in low serum (0.5% FBS) medium. DMSO (0.1%) and VEGF (20 ng/mL) served as negative and positive controls, respectively. In accordance with the manufacturer’s protocol, plates were incubated for an additional 48 h and cellular proliferation was assessed by the MTT, which is widely used to observe the growth of cell. The spectrophotometric absorbance of each well was measured. The wavelengths used to measure absorbance of the formazan product were 570 nm and 630 nm. The results were expressed as the percentage of proliferating cells.

### 3.5. Migration Assays

HUVEC migration assays were performed using the wound healing method. The HUVECs (3 × 10^5^ cells) were seeded into each well of a 24-well plate and incubated with complete medium at 37 °C and 5% CO_2_. After 24 h of incubation, cells were starved for additional 24 h by low serum (0.5% FBS) medium. The HUVECs were then scraped away horizontally in each well using a P100 pipette tip. Three randomly selected views along the scraped line were photographed on each well using an Olympus ix53 microscope (Olympus, Tokyo, Japan) and the CCD camera attached to the microscope at 10× magnification. The medium was then changed to fresh low serum (1% FBS) medium with compounds **1** and **5**–**7** (50 μM) or with DMSO. After incubation (0 h, 12 h, 24 h, 36 h and 48 h), another set of images were taken by the same method. Image analysis for signs of migration was performed by Metamorph Imaging Series (Molecular Devices, LLC., San Jose, CA, USA). The average scraped area of each well under each condition was measured and subtracted from that of the before-treatment condition. Data are expressed as percentage wound closure relative to the wound closure area in the control medium. The wound closure area of the control cells was set at 100%.

### 3.6. Invasion Assay

HUVEC invasion assay was carried out following previous methods [13]. Briefly, the effect of compounds **1** and **5**–**7** on HUVEC invasion was measured using the 10 mm tissue culture insert (transwell permeable supports, Corning Incorporated, Tewksbury, MA, USA) with polycabonate membarane (8 mm pores) and 24-well companion plate. The upper side and lower side of the membrane were pre-coated with 1:30 (*v/v*) of Matrigel (Corning Incorporated, Tewksbury, MA, USA). The HUVECs were resuspended in low serum (1% FBS) medium and seeded onto the culture inserts at 5 × 10^4^ cells per insert in triplicate. They were then deposited into the 24-well companion plate with 500 μL of low serum (1% FBS) medium containing compounds (50 μM) in the presence. In addition, the wells of the companion plate, containing DMSO (0.1%), served as a vehicle control. The inserts were removed after 8 h of incubation and were then washed with PBS. Non-invasive cells on the upper surface of the membrane were removed by wiping with cotton swabs. The inserts were fixed in paraformaldehyate, stained with DAPI and mounted on a microscope and a CCD camera. Following this, HUVECs per insert were examined with the software Metamorph Imaging Series (Molecular Devices, Tokyo, Japan).

### 3.7. Zebrafish Assay of Compound ***7***

Zebrafish embryos were used to examine the effect of different compounds on embryonic angiogenesis. Compound **7** was added to embryo water from 24 hpf. Zebrafish embryos were generated by natural pairwise mating and raised at 28.5 °C in embryo water. Embryos were maintained in embryo water at 28 °C. Three embryos were placed into each well of a 96-well plate containing 200 μL embryo water with or without the drug. The blood vessel development using an inverted Olympus DP70 epifluorescence microscope (Olympus, Tokyo, Japan). Because the fish embryo receives nourishment from an attached yolk ball for the duration of the experiment, no additional maintenance was required during the duration of the experiments. After 72 hpf, the embryos were anesthetized using 0.05% 2-phenoxyethanol in embryo water, and each embryo was examined for the presence of ectopic vessels in the subintestinal vessel plexus (SIV). The experiments of zebrafish were conducted according to the guidelines for animal care and use of China and were approved by the animal ethics committee of the Chinese Academy of Medical Science (Beijing, China).

PTK787 is frequently used as angiogenesis inhibitors [18]. In order to test the effect of compound **7** on damaged zebrafish, we evaluated a quantitative assay in transgenic zebrafish using angiogenesis inhibitor PTK787. The 24 hpf embryos were cultured and collected. The inhibitor, PTK787 (0.03 μg/mL) was added into embryo water and the embryo were cultured 24 h. Subsequently, compound **7** was added into embryo water afer removing the PTK787 and cultured for 24 h. After 72 hpf, the embryos were anesthetized using 0.05% 2-phenoxyethanol in embryo water, and each embryo was examined for the presence of ectopic vessels in the subintestinal vessel plexus (SIV).

### 3.8. Statistical Analysis

Statistical analysis was performed using SPSS Statistics 21 software (IBM corporation, Armonk, NY, USA). Survival curves were analyzed by the life table method and evaluation of the effects of compounds on the mean survival time was done by the Wilcoxon rank sum test. All the curves and column diagrams were drawn using GraphPad Prism 6 software (GraphPad Software, Inc., San Diego, CA, USA). Data are expressed as the mean ± SEM. Statistical comparisons between groups were performed using one-way ANOVA followed by Dunnett’s *t*-test using non-treatment as the control group. *p* < 0.05 was considered statistically significant.

## 4. Conclusions 

In summary, seven compounds have been designed and synthesized to evaluate the proliferation, migration and invasion of HUVECs by MTT assays, based on the lead compound **1**, which was demonstrated significantly stimulate angiogenesis both in vivo and in vitro. Among these analogues, compounds **5**–**7** possess remarkable proliferations, migrations and invasions of HUVECs compared with the lead compound 1. The results show that hydrophilic, alkaline group, l-Tyrosine and d-Proline fragment substituting for L-alanine may greatly contribute to proliferation of HUVECs. To our surprise, compound **7** exerted a significant effect on HUVECs, which revealed that the l-Arg-d-Val fragment resembles Lys(Boc)-l-Ala-OH in terms of cytoactivity. With its good proliferation, compound **7** can promote angiogenesis in zebrafish and can repair blood vessels in PTK787-induced zebrafish at a low concentration. These small molecular peptides could be easily prepared compared the macromolecule proteins. Because of the briefness of their strutures, they would eventually develop into a promising drug candidate for the treatment of damage repair and related diseases.

## Figures and Tables

**Table 1:** Values EC_50_ (μM) of compounds with respect to HUVEC proliferation**.**

Compounds	EC_50_ (μM)
**1**	1.31 ± 0.0926
**2**	57.55 ± 6.10
**3**	>200.00
**4**	76.02 ± 0.205
**5**	1.00 ± 0.002
**6**	1.00 ± 0.0005
**7**	0.88 ± 0.0972
**8**	1.33 ± 0.201

## Supplementary Materials

The following are available online, Figure S1: ^1^H NMR (MeOD, 500 MHz) of Compound **1**, Figure S2: ^13^C NMR (MeOD, 125 MHz) of Compound **1**, Figure S3: ^1^H NMR (MeOD, 500 MHz) of Compound **2**, Figure S4: ^13^C NMR (MeOD, 125 MHz) of Compound **2**, Figure S5: ^1^H NMR (MeOD, 500 MHz) of Compound **3**, Figure S6: ^13^C NMR (MeOD, 125 MHz) of Compound **3**, Figure S7: ^1^H NMR (MeOD, 500 MHz) of Compound **4**, Figure S8: ^13^C NMR (MeOD, 125 MHz) of Compound **4**, Figure S9: ^1^H NMR (MeOD, 500 MHz) of Compound **5**, Figure S10: ^13^C NMR (MeOD, 125 MHz) of Compound **5**, Figure S11: ^1^H NMR (MeOD, 500 MHz) of Compound **6**, Figure S12: ^13^C NMR (MeOD, 125 MHz) of Compound **6**, Figure S13: ^1^H NMR (MeOD, 500 MHz) of Compound **7**, Figure S14: ^13^C NMR (MeOD, 125 MHz) of Compound **7**, Figure S15: ^1^H NMR (MeOD, 400 MHz) of Compound **8**, Table S1: Purities and retention times of all tested compounds, Table S2: HPLC chromatography of compound **1**, Table S3: HPLC chromatography of compound **2**, Table S4: HPLC chromatography of compound **3**, Table S5: HPLC chromatography of compound **4**, Table S6: HPLC chromatography of compound **5**, Table S7: HPLC chromatography of compound **6**, Table S8: HPLC chromatography of compound **7**, Table S9: HPLC chromatography of compound **8**.
