# Supplementary material for: Studies on the Design and Synthesis of Marine Peptide Analogues and Their Ability to Promote Proliferation in HUVECs and Zebrafish
Source: Molecules. 2018 Dec 25;24(1):66. doi: 10.3390/molecules24010066 (PMC6337321; doi:10.3390/molecules24010066)

Supporting Information

Studies on Design, Synthesis of Marine Peptide analogues and their Promoting Proliferations on HUVECs and Zebrafish

Yinglin Zheng [1], Yichen Tong [1], Xinfeng Wang [1], Jiebin Zhou [1] and Jiyan Pang [1,*]

Jiyan Pang: Email: cespjy@mail.sysu.edu.cn;

TABLE OF CONTENTS

NMR spectra of compound **1**	3
NMR spectra of compound **2**	4
NMR spectra of compound **3**	5
NMR spectra of compound **4**	6
NMR spectra of compound **5**	7
NMR spectra of compound **6**	8
NMR spectra of compound **7**	9
NMR spectra of compound **8**	10
Purities and retention times of all tested compounds	11-13

Figure S1: ^{1}H NMR (MeOD, 500 MHz) of Compound **1**

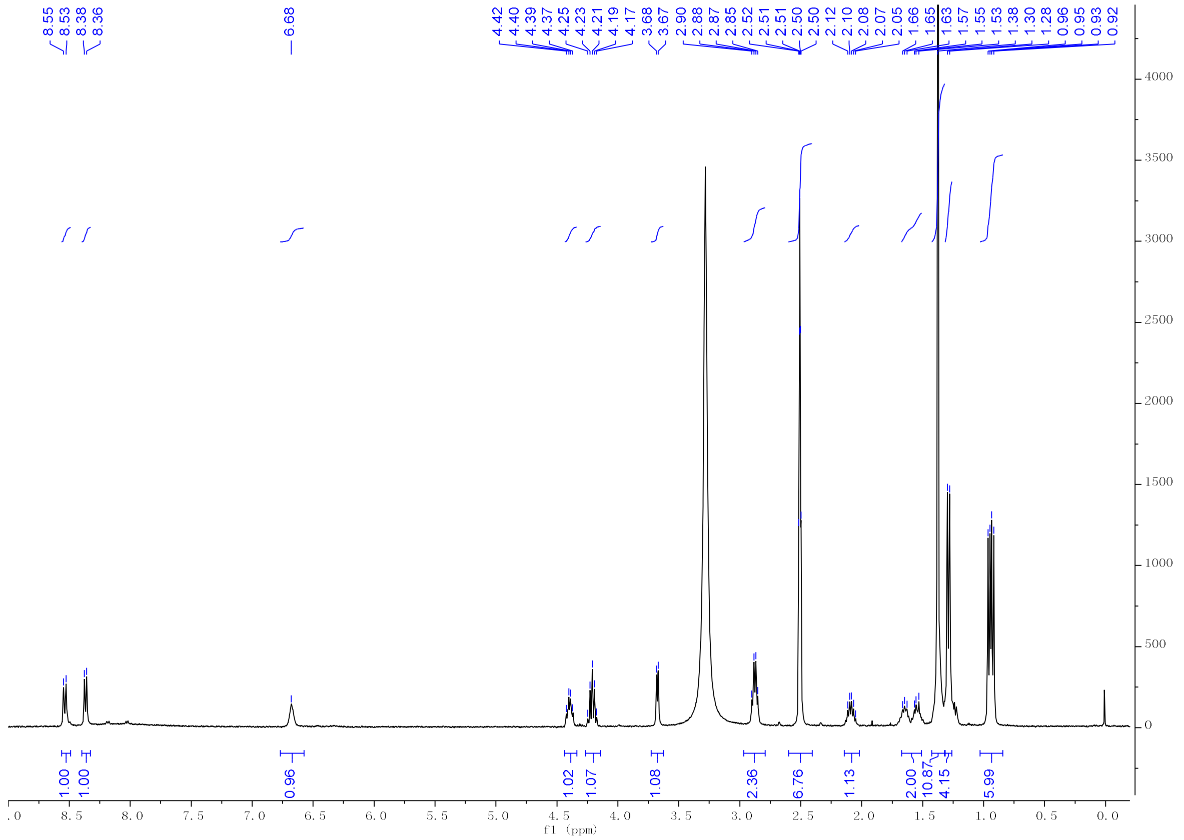

Figure S2: ^{13}C NMR (MeOD, 125 MHz) of Compound **1**

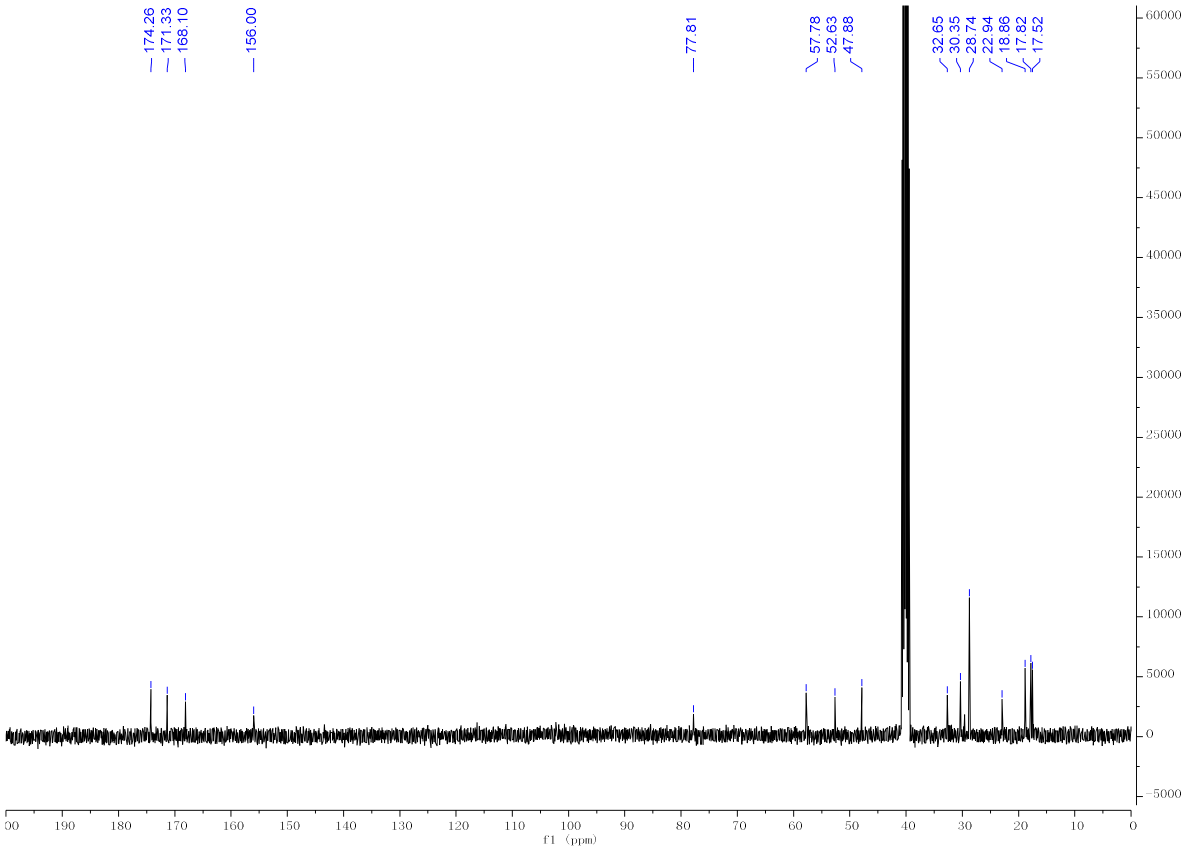

Figure S3: ^{1}H NMR (MeOD, 500 MHz) of Compound **2**

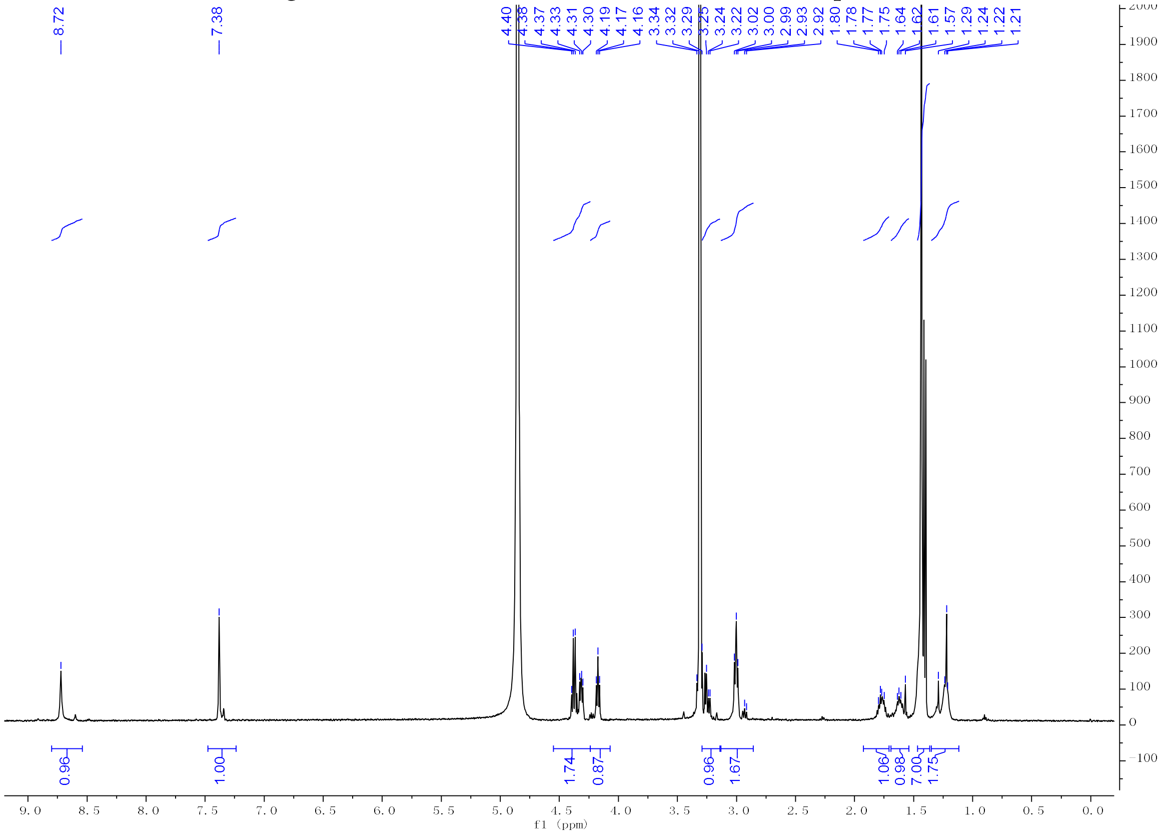

Figure S4: ^{13}C NMR (MeOD, 125 MHz) of Compound **2**

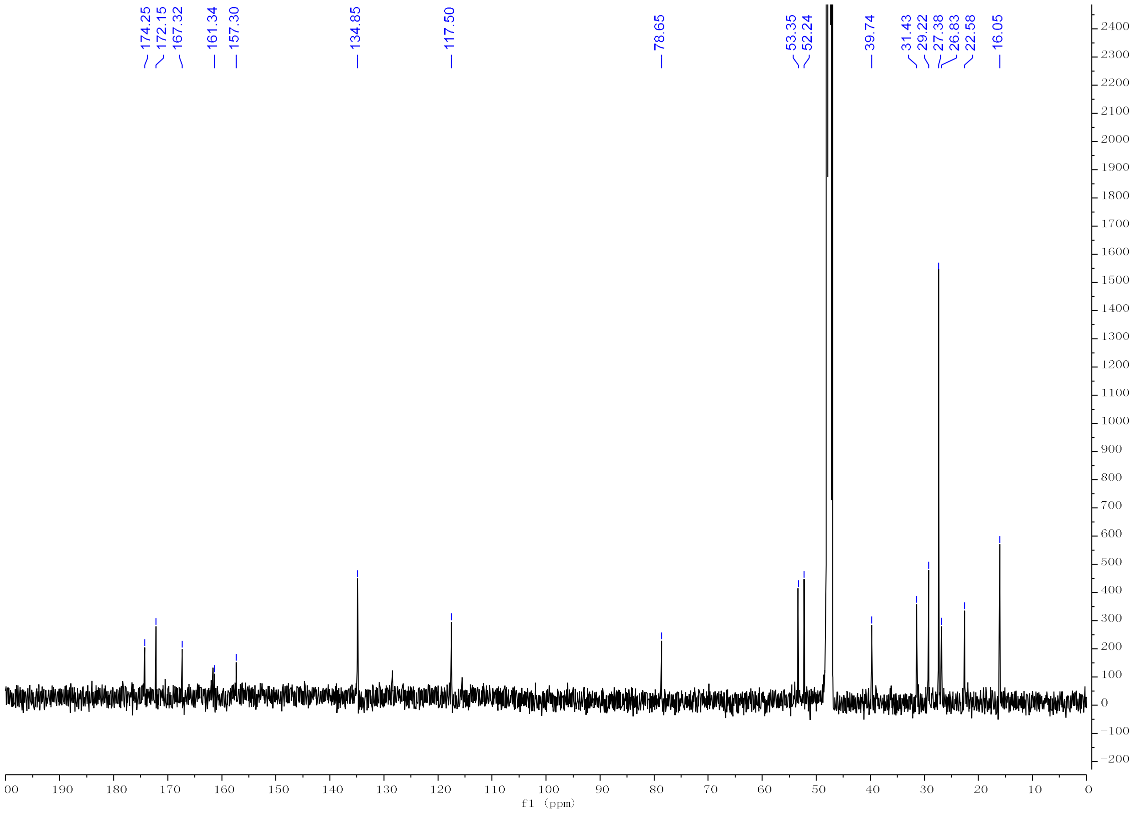

Figure S5: ^{1}H NMR (MeOD, 500 MHz) of Compound **3**

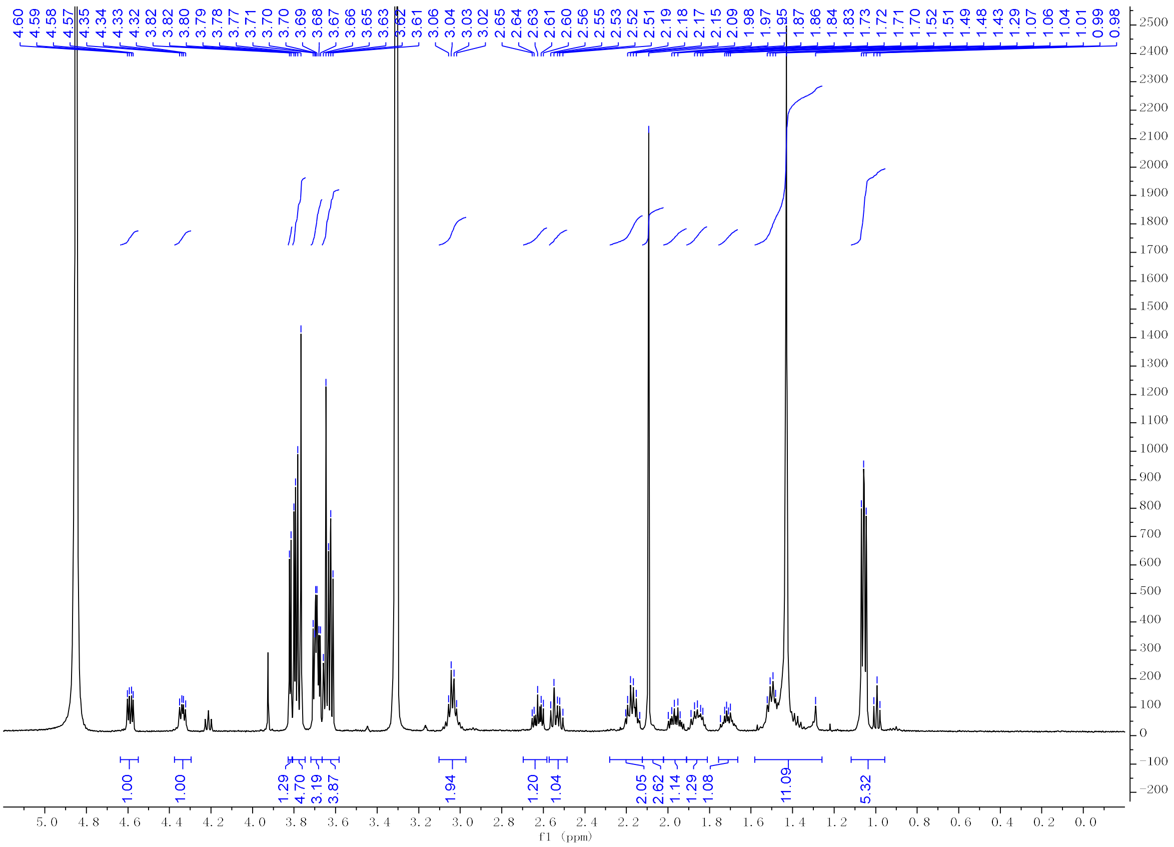

Figure S6: ^{13}C NMR (MeOD, 125 MHz) of Compound **3**

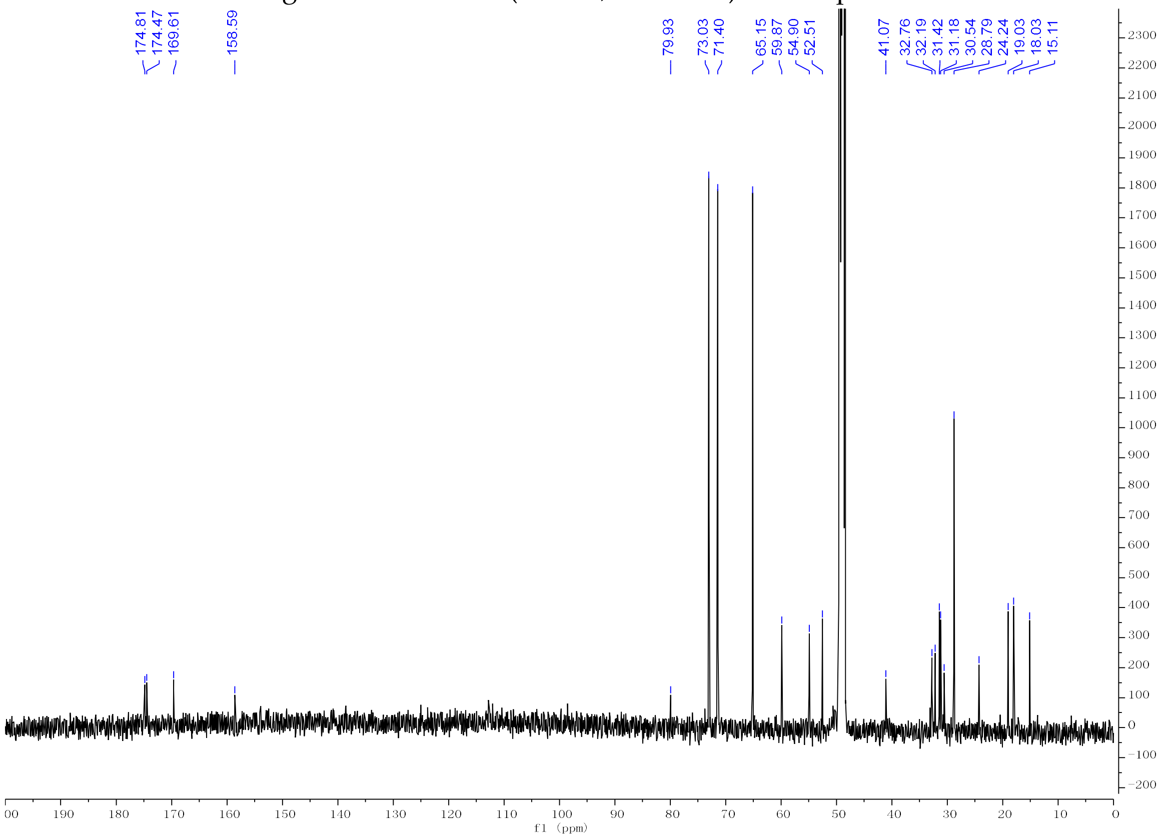

Figure S7: ^{1}H NMR (MeOD, 500 MHz) of Compound **4**

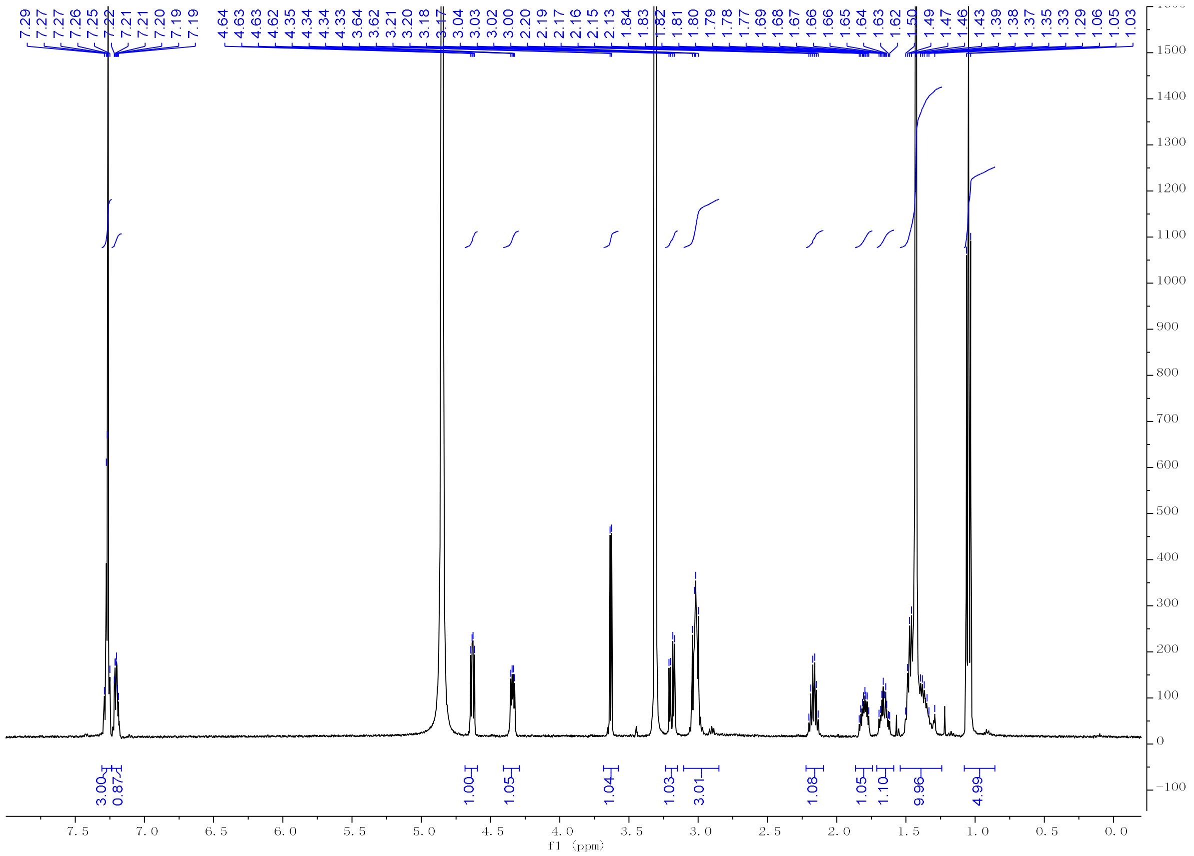

Figure S8: ^{13}C NMR (MeOD, 125 MHz) of Compound **4**

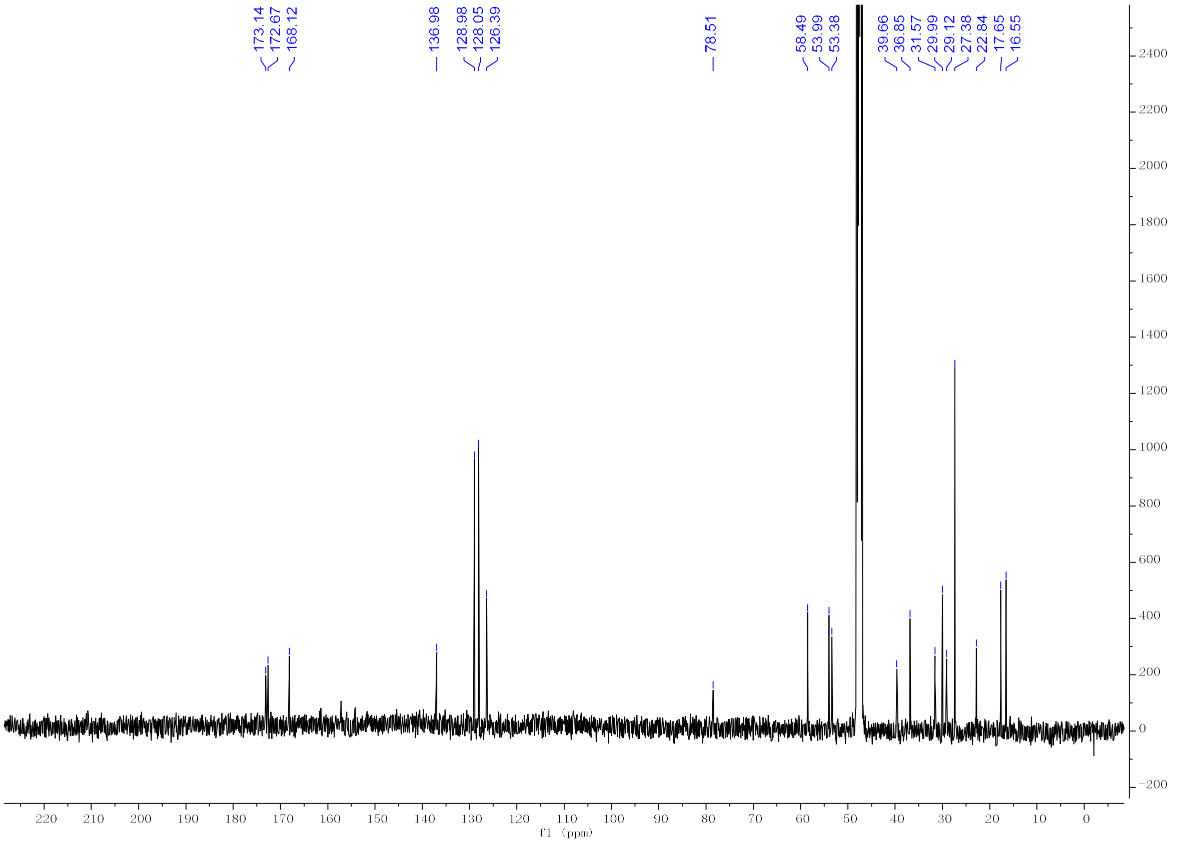

Figure S9: ^{1}H NMR (MeOD, 500 MHz) of Compound **5**

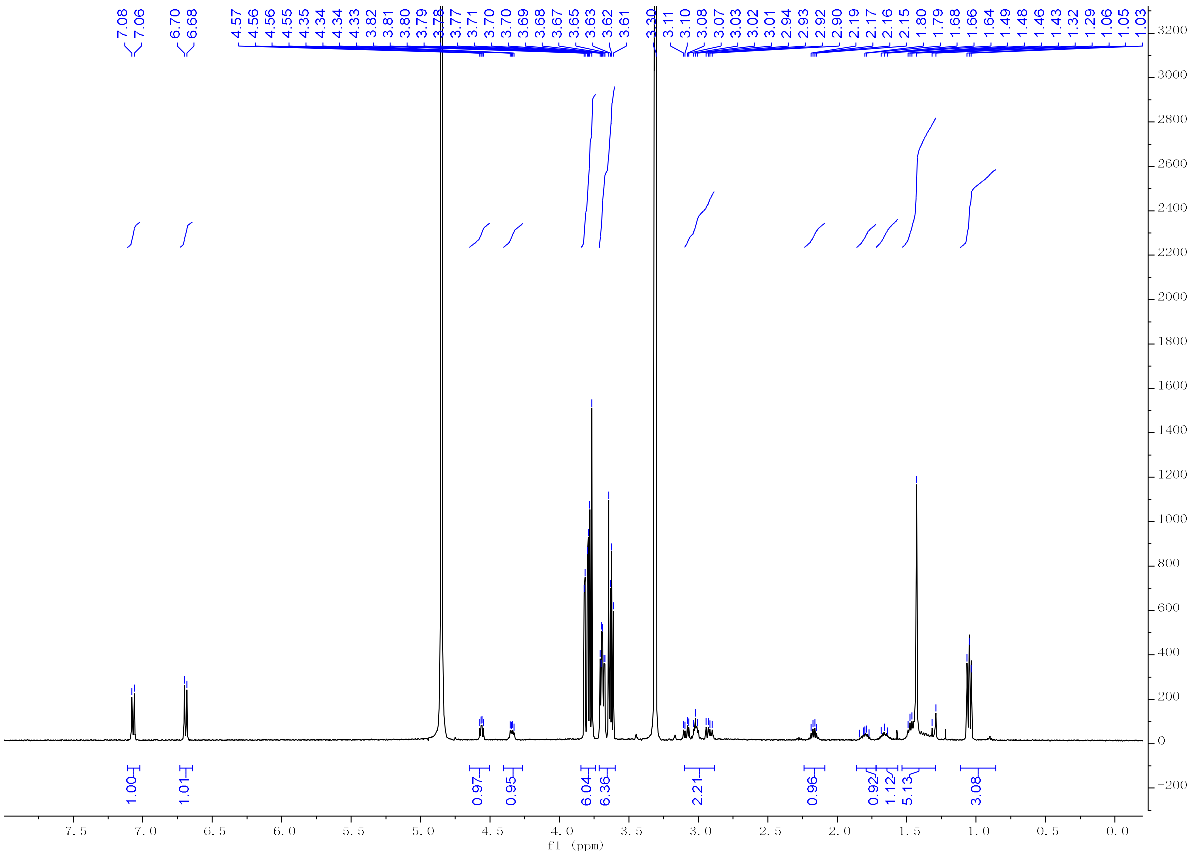

Figure S10: ^{13}C NMR (MeOD, 125 MHz) of Compound **5**

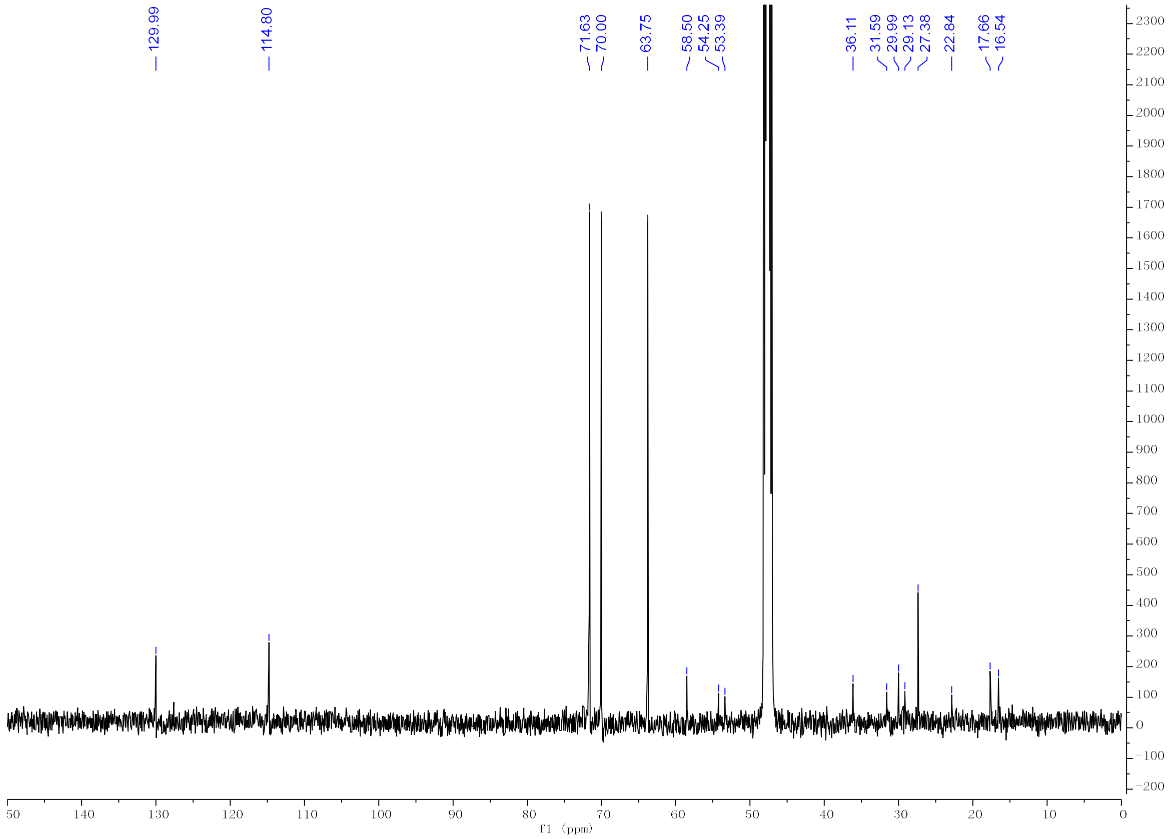

Figure S11: ^{1}H NMR (MeOD, 500 MHz) of Compound **6**

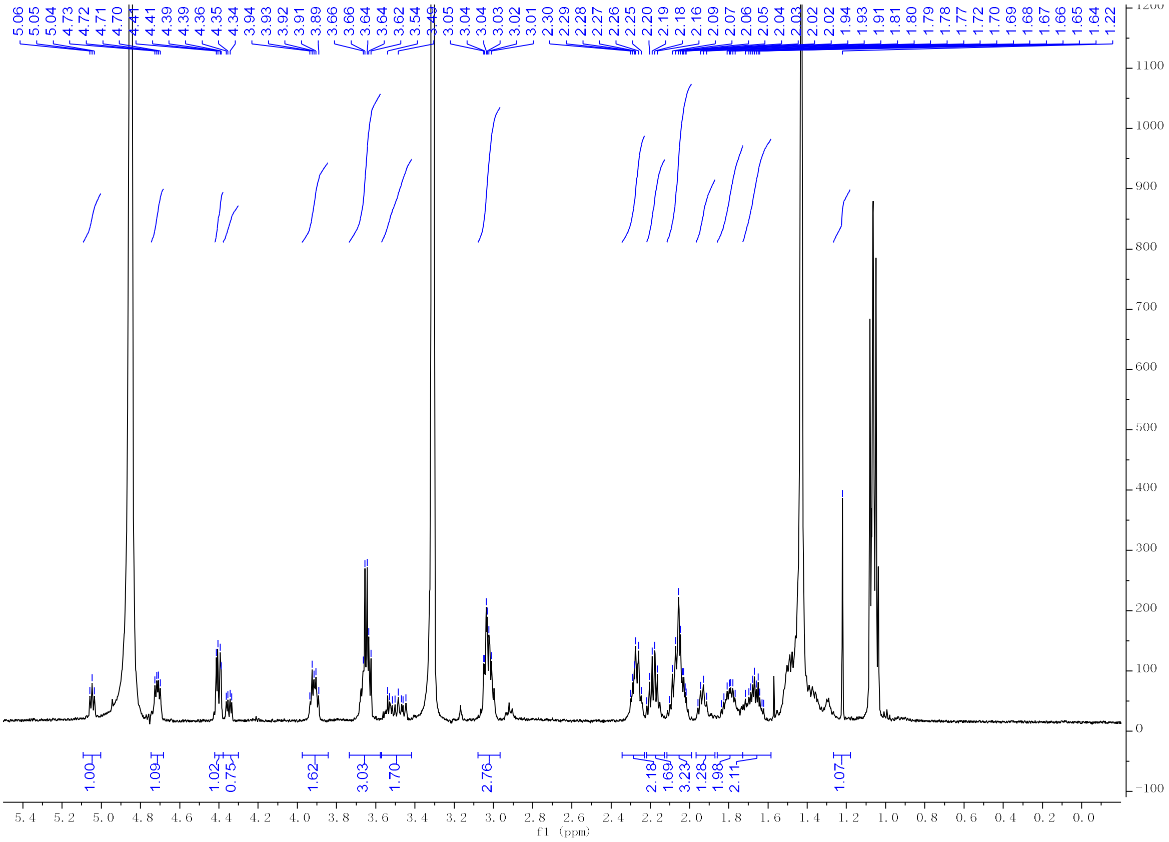

Figure S12: ^{13}C NMR (MeOD, 125 MHz) of Compound 6

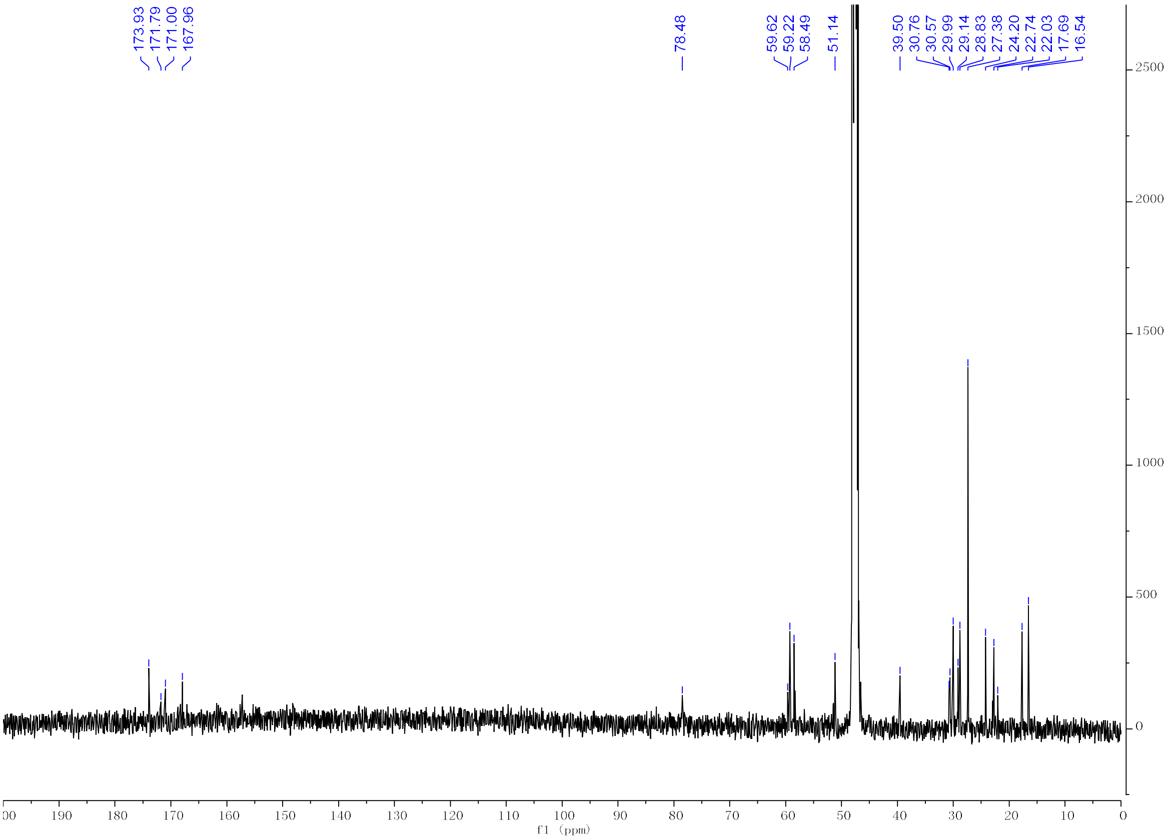

Figure S13: ^{1}H NMR (MeOD, 500 MHz) of Compound **7**

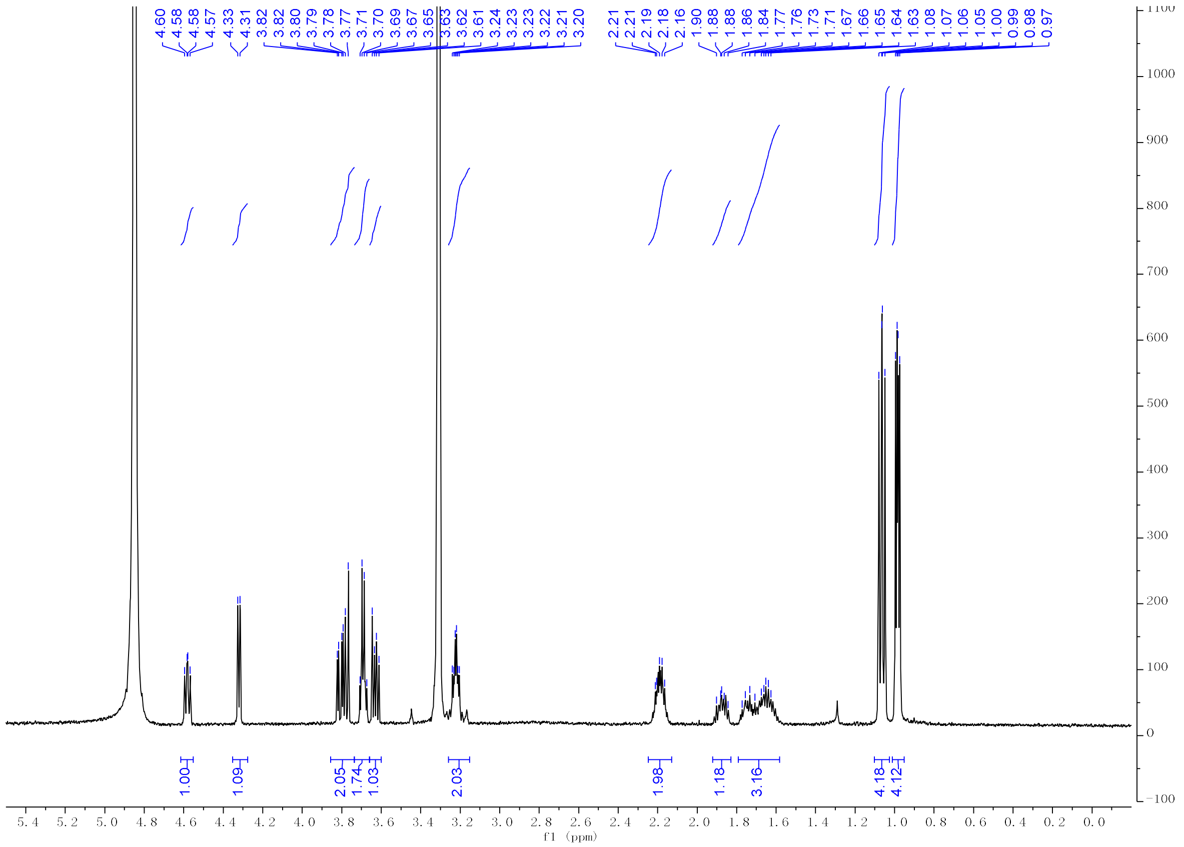

Figure S14: ^{13}C NMR (MeOD, 125 MHz) of Compound **7**

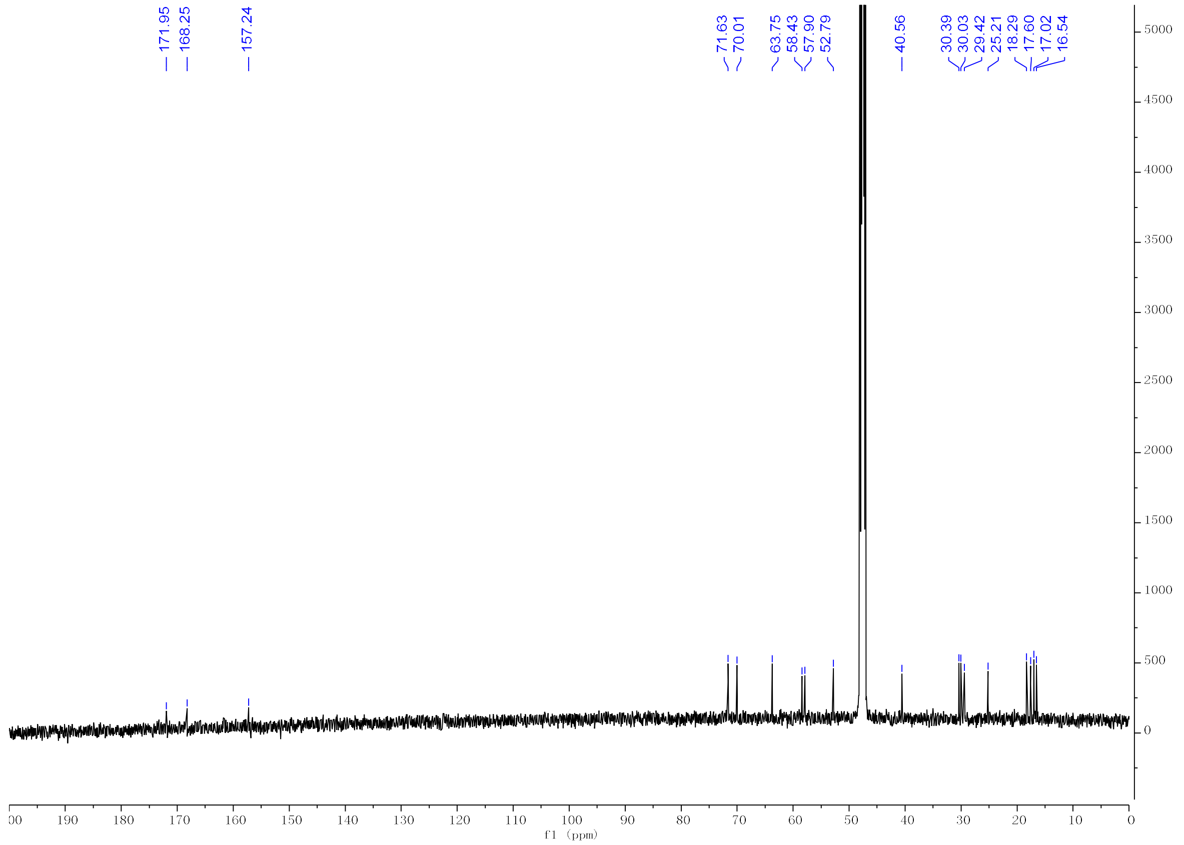

Figure S15: ^{1}H NMR (MeOD, 400 MHz) of Compound **8**

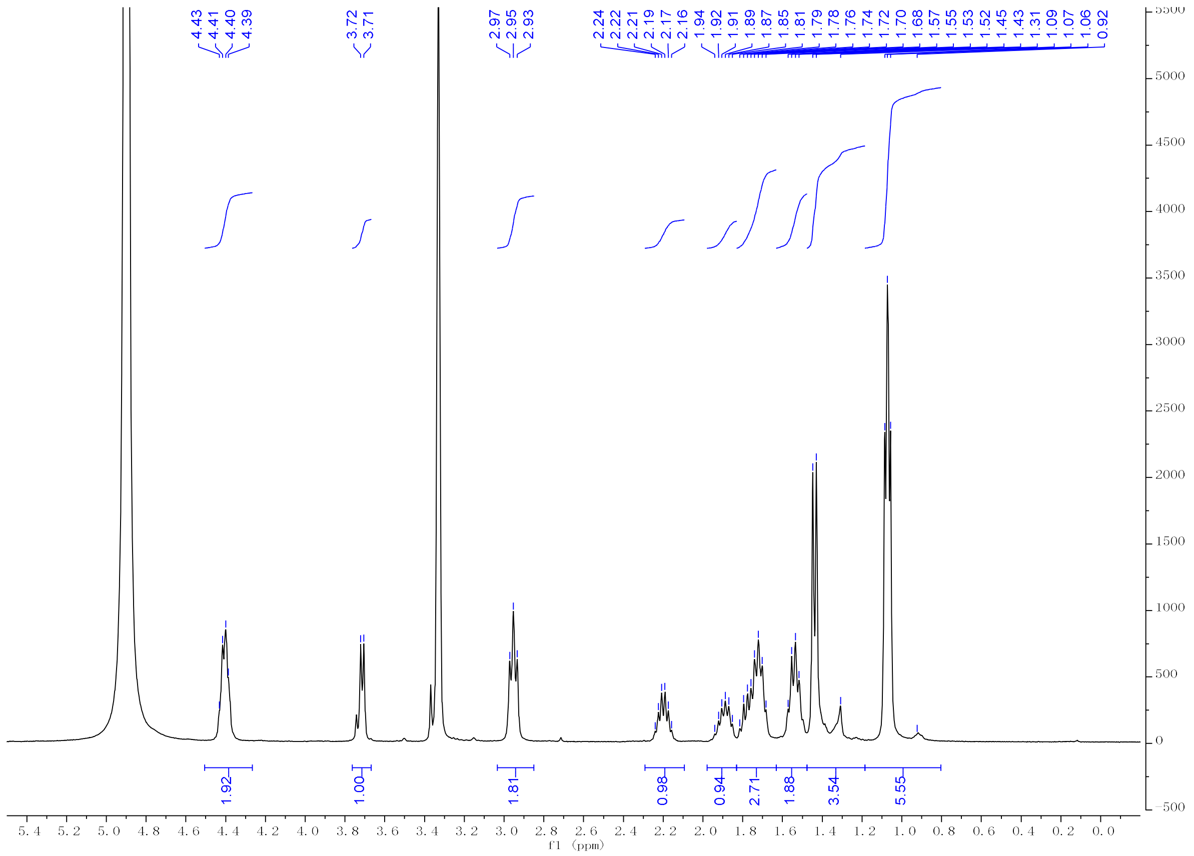

The purities of all tested compounds were at least 95% and were determined by HPLC equipped with an UV detector at 210nm/254nm/280nm and 4.6*250mm(5μm) Chiralcel OD-H chiral column at 20℃. Ultrasonic degassing to all bought HPLC grade solvents for 30 minutes were done before used. Purity analysis were run with elution conditions. Elution condition: water: acetonitrile 40:60 (v/v), flow rate = 1 mL/min. It was stated if 0.1% acetic acid was added to the water. Table 1 is a summary of the purities and retention times of compounds

Table 1: Purities and retention times of all tested compounds.

Comp.	Elution condition	
	purity	Retention time / min
1	97.09	3.169
2	95.41	2.098
3	99.87	5.371
4	96.61	2.888
5	98.17	3.977
6	95.14	4.216
7	98.95	2.337
8	95.27	2.553

* the water phase was added 0.1% acetic acid.

Table 2: HPLC chromatography of compound **1**

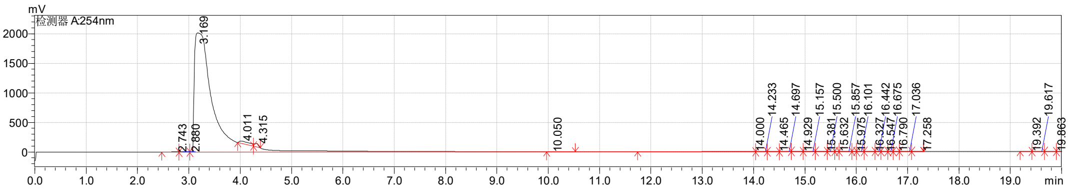

Table 3: HPLC chromatography of compound **2**

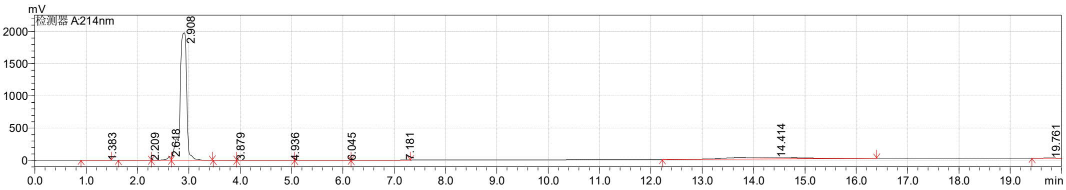

Table 4: HPLC chromatography of compound **3**

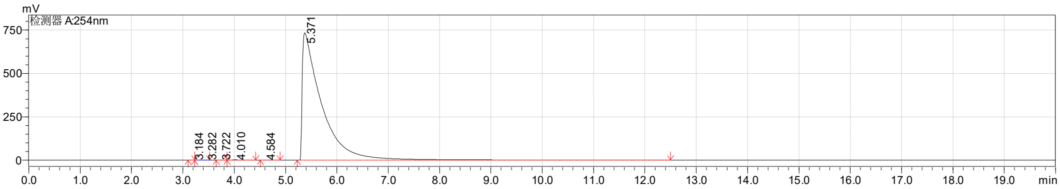

Table 5: HPLC chromatography of compound **4**

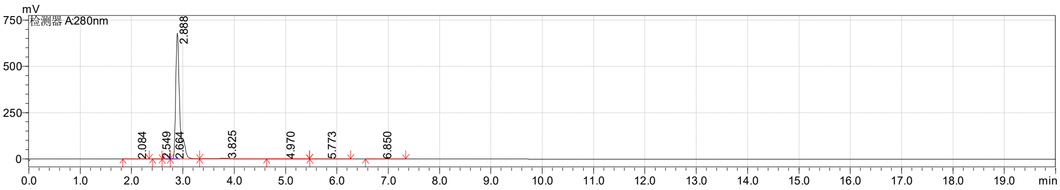

Table 6: HPLC chromatography of compound **5**

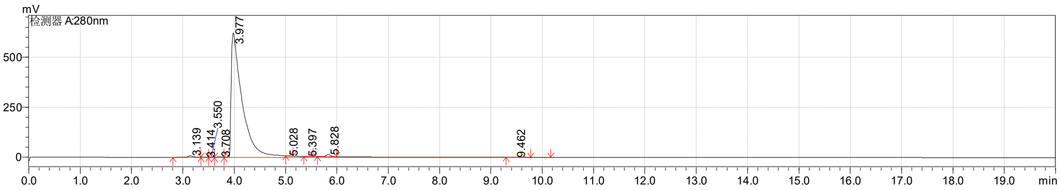

Table 7: HPLC chromatography of compound **6**

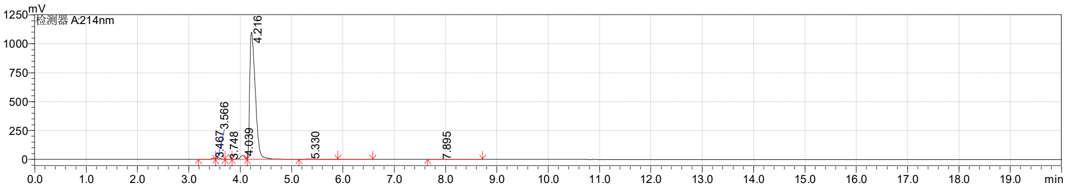

Table 8: HPLC chromatography of compound **7**

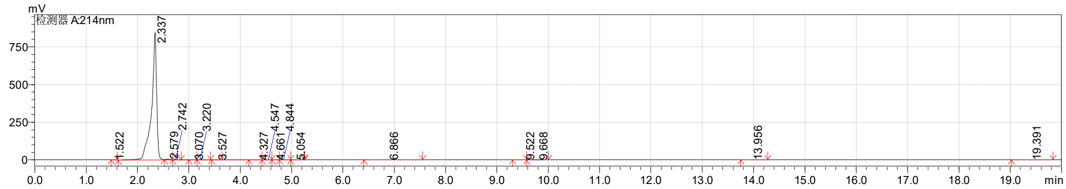

Table 9: HPLC chromatography of compound **8**

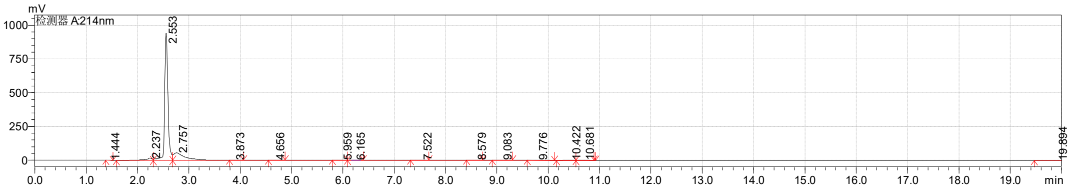

Supplement: Supplementary file 1 [file molecules-24-00066-s001.pdf]
